# Development of pediatric simulation-based education – a systematic review

**DOI:** 10.1186/s12912-023-01458-8

**Published:** 2023-08-28

**Authors:** EunJoo Kim, SungSook Song, SeongKwang Kim

**Affiliations:** 1 Department of Nursing, Gangneung-Wonju National University, 150, Namwon-ro, Heungop- myeon, Wonju-si, 26403 Gangwon-do Republic of Korea; 2 Department of Nursing, INHA University, 313, Docbae-ro, Michuhol-gu, Incheon, 22188 Republic of Korea

**Keywords:** Pediatrics, Simulation, Systematic review, Simulation-based education, Scenario, Validation, Reliability

## Abstract

**Background:**

This systematic literature review explored the general characteristics, validation, and reliability of pediatric simulation-based education (P-SBE).

**Methods:**

A literature search was conducted between May 23 and 28 using the PRISMA guidelines, which covered databases such as MEDLINE, EMBASE, CINAHL, and Cochrane Library. In the third selection process, the original texts of 142 studies were selected, and 98 documents were included in the final content analysis.

**Results:**

A total of 109 papers have been published in the ten years since 2011. Most of the study designs were experimental studies, including RCT with 76 articles. Among the typologies of simulation, advanced patient simulation was the most common (92), and high-fidelity simulation was the second most common (75). There were 29 compatibility levels and professional levels, with 59 scenarios related to emergency interventions and 19 scenarios related to communication feasibility and decision making. Regarding the effect variable, 65 studies confirmed that skills were the most common. However, validity of the scenarios and effect variables was not verified in 56.1% and 67.3% of studies, respectively.

**Conclusion:**

Based on these findings, simulation based-education (SBE) is an effective educational method that can improve the proficiency and competence of medical professionals dealing with child. Learning through simulation provides an immersive environment in which learners interact with the presented patient scenario and make decisions, actively learning the attitudes, knowledge, and skills necessary for medical providers. In the future, it is expected that such research on SBE will be actively followed up and verified for its validity and reliability.

## Background

### Rationale for the study

Simulation-based education (SBE) is not a technology, but a learner-centered pedagogical method based on learning theories [1]. The greatest benefit of SBE is that it enables repeated training in a safe environment resembling an actual hospital setting [2]. For example, students can experience cases in which they cannot be directly involved in a clinical setting, such as providing care for a psychiatric patient exhibiting dangerous behaviors or end-of-life care for patients and their families [1]. Moreover, training that requires a more realistic setting, such as dissection, can be performed using immersive virtual reality [3]. As shown here, SBE can be designed with the desired scenario contents based on the learning objectives, and patient information and simulators can be varied to provide different SBE [1].

Simulation-based education helps nursing students to establish their professional identity by experiencing the roles of a nurse in advance [4], and question and-answer sessions and discussions with the instructor during debriefing after the training allows students to engage in self-reflection, through which they can integrate their learned materials and translate them into practice [5]. Due to these benefits, SBE supplements clinical practicum across all topics. Recently, it’s especially advised for situations where students can’t directly interact, like pediatric vaccinations, asthma treatments, and mother-infant cases [1].

As a result of the coronavirus disease 2019 (COVID-19) pandemic that struck the world in 2019, clinical practicum was either suspended or stopped for patient and student safety, and students expressed anxiety about potentially contracting the infection from patients or other students during clinical practicum [6], further highlighting the need for SBE. Moreover, pediatric nursing clinical practicum is very challenging in the Republic of Korea (ROK) compared with other clinical practicums. The ROK is one of the countries with the lowest fertility rates, and it has the most quickly declining cumulative birth rate and total fertility rate among 37 organization for economic cooperation and development (OECD) countries, with an average annual drop of 3.1%. In addition, the number of neonates has dropped dramatically from 490,000 to 2012 to 260,000 in 2021 [7]. Moreover, the number of high-risk neonates vulnerable to infection and injury is on the rise, from 18,232 to 1995 to 30,462 in 2015 [7], which further hinders students from encountering divers even if clinical practicum courses are offered.

A systematic review of studies that conducted a cost analysis for SBE reported that the most common topic—following surgery cases—was pediatrics and obstetrics and gynecology, and that most studies were conducted in low-income countries, with common topics being neonatal and maternal health care, such as “Helping Babies Breathe” (HBB) and “Essential Newborn Care” (ENC) [2]. As shown here, pediatric health is a very important topic of SBE not only in countries with low fertility rates but also in low-income countries. Providing pediatric nursing clinical practicum is very challenging due to the declining number of newborns, increasing incidence of high-risk births, and high cost associated with SBE.

To address these issues, a growing number of studies have evaluated the effects of SBE; however, the types of SBE studied vary widely, and the validity and reliability of scenarios and contents of SBE have not been adequately evaluated. Furthermore, diverse outcome measures have been used and standardized instruments are lacking [4, 8, 9]. The validity of the simulation was described as the degree to which the simulation accurately represented the target task, and the reliability of the simulation was described as the degree to which simulation education was measured using the same method each time the same participants received education under the same conditions [10]. Because simulation is an educational method that enables nursing educators to facilitate and assess learners’ clinical competencies [1], educators must develop valid and reliable scenarios and assess learners using standardized instruments.

There are several types of simulators available, including standard patients, high-fidelity simulators, low-fidelity simulators, and partial task simulators. Instructors choose the type of simulator based on the objectives of SBE. Consequently, the use of an ineffective simulator may curtail the effectiveness of education [1].

As shown here, past systematic reviews of studies on SBE have primarily conducted technical analyses of educational methods and target populations, with a lack of systematic reviews on the contents of SBE. In this context, we conducted a systematic review to examine the characteristics of pediatric simulation-based education (P-SBE) and evaluate the validity and reliability of the development process. The findings of this study will shed light on the direction of future SBE programs and interventions and establish criteria for validity and reliability evaluations of simulation scenarios and programs.

### Research questions

This study was a systematic review of past studies that have developed and evaluated the effects of P-SBE. The findings of this study will be used as criteria for evaluating the validity and reliability of future P-SBE. The specific research questions were as follows:

1. Review the characteristics of studies that developed and evaluated the effects of P-SBE.
2. Identify the characteristics of scenarios used in P-SBE.
3. Evaluate the validity and reliability of the process of developing P-SBE.
4. Evaluate the validity and reliability of instruments used to assess the effects of P-SBE.

## Methods

### Study design

This study conducted a systematic review of P-SBE, specifically examining the general characteristics of the studies, topics of education, simulation methods, reliability and validity of simulation, and dependent variables. The key question selection, literature selection based on inclusion and exclusion criteria, data extraction, setting of scope of literature search and search databases, quality appraisal, and risk of bias assessment were performed in accordance with the Preferred Reporting Items of Systemic Reviews and Meta-Analysis (PRISMA) 2020 statement [11] and 2022 Cochrane Handbook for Systematic Reviews of Interventions version 6.3 [12], and data were analyzed. We classified the characteristics of literature based on typology, referencing the definition of “Simulation Typologies/Modalities” provided by Palaganas et al. in 2020 [13].

### Key questions and selection criteria

The key questions of this study were: “What is the construction of P-SBE?” and “What aspects are assessed in P-SBE?”. The specific inclusion criteria were as follows:1) studies that developed a simulation program or scenario, 2) pediatric scenarios, and 3) health and health care-related scenarios (not necessarily in clinical settings, but including events such as traffic accidents, bee stings, bicycle accidents, daily life shocks, etc., these criteria were included in the third round of literature screening). The exclusion criteria were as follows:1) studies on non-human simulations (even if they are related to pediatrics, studies about the development of simulators, etc., were excluded), 2) non-pediatric scenarios, and 3) studies on non-human simulations (even if they are related to pediatrics, studies about the development of simulators, etc., were excluded). The search strategy was established based on the PICO-SD framework for non-Korean databases: “(simulat* or scenario*) and (pediatric or child or children or baby or newborn or infant or kid*) and (valid* or reliab*).

### Literature search and selection process

Two researchers independently performed a literature search. The search was conducted from May 23, 2022, to May 28, 2022. The MEDLINE, EMBASE, CINAHL, and Cochrane Library databases were selected according to the PRISMA statement. An advanced search was performed based on the participants, intervention, comparison, outcome, and study design (PICO-SD) framework. In addition, a search was performed using Google Scholar to include as many gray articles as possible.

**Fig. 1:**
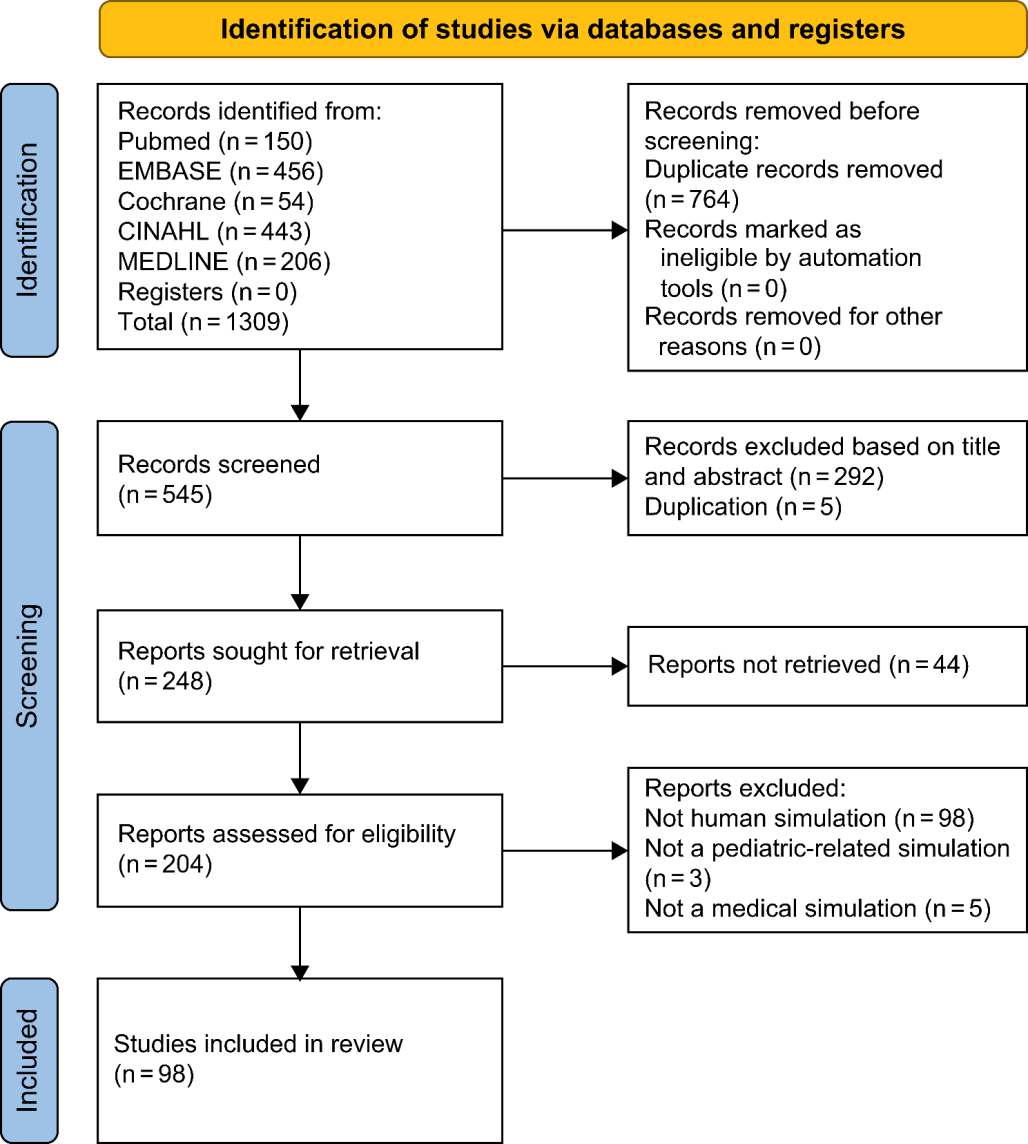
PRISMA flow diagram

The criteria for the initial screening were set according to the PICO-SD framework. We did not define a specific participant population and included healthcare providers, nurses, and nursing students. As for the intervention, all P-SBE programs were included. The outcome variables were not specified. For the study design, we included all studies that observed effective outcomes after administering an SBE program, and studies that presented data for the validity and reliability of the scenario and instruments. A total of 1,309 studies were selected during the initial screening and 764 duplicates were excluded. In the second round of screening, the titles and abstracts of 545 studies were independently reviewed by three researchers based on the PCIO-SD criteria. In total, 292 studies were excluded. In the third round of screening, the full texts of the selected studies were obtained, and full texts of 253 studies were available. Of these, 111 studies did not meet the inclusion criteria and were excluded. From the resulting 142 studies, 44 were excluded from the content analysis because they were proceedings and did not show the details of the scenarios. Thus, 98 studies were included in the content analysis. Each researcher independently evaluated the quality of the papers using the Mixed Methods Appraisal Tool (MMAT), 2018 [14]. Only papers with moderate-to high-quality ratings were included in the review. Any disagreements among the researchers during this process were resolved by discussion. If the selected studies did not state the detailed study methodology, the researchers described it upon discussion (Fig. 1).

### Data analysis

The 98 included studies were written as case reports, and qualitative analysis was performed using Excel 2016 software. The case reports contained information about general characteristics (authors, year, title, and country), study characteristics (study design, participant characteristics, simulator type, scenario topic, scenario reliability, and validity), and outcome characteristics (dependent variables, instruments used to measure dependent variables, and reliability and validity of dependent variables).

### Study results

#### Characteristics of the studies

Table 1 presents the general characteristics of the included studies. A total of 142 studies pertinent to P-SBE were identified. Fifteen (10.6%) were published between 2001 and 2010, and 109 (76.8%) were published in the subsequent ten years, showing a more than seven-fold increase. The greatest number of studies were conducted in the United States (n = 62, 43.7%), followed by Canada (n = 17, 12.0%). Experimental studies, including randomized controlled trials (RCTs), were the most common (n = 76, 53.5%), followed by developmental studies, including simulation development (n = 58, 40.85%). According to simulation typology, advanced patient simulation was the most common (n = 92, 64.8%). Most studies used high-fidelity simulation only (n = 75, 52.8%), followed by video-based simulation, and four studies used VR simulation.

**Table 1:** General Characteristics of the selected studies (n = 142)

Characters	Categories	Subcategories	n	Percent
Year of publish	Before 2000 (n = 2)	1996	1	0.7
		2000	1	0.7
	From 2001 to 2010 (n = 15)	2003	1	0.7
		2007	2	1.4
		2008	2	1.4
		2009	2	1.4
		2010	8	5.6
	From 2011 to 2020 (n = 109)	2011	6	4.2
		2012	14	9.9
		2013	8	5.6
		2014	14	9.9
		2015	7	4.9
		2016	10	7.0
		2017	8	5.6
		2018	20	14.1
		2019	15	10.6
		2020	7	4.9
	From 2021 to 2022 (n = 16)	2021	10	7.0
		2022	6	4.2
Nation of sample	North America (n = 79)	USA	62	43.7
		Canada	17	12.0
		USA/Botswana	1	0.7
	South America (n = 6)	Brazil	4	2.8
		Colombia	1	0.7
		Peru	1	0.7
	Europe (n = 24)	UK	10	7.0
		Germany	2	1.4
		Switzerland	2	1.4
		Switzerland/ Germany	2	1.4
		Sweden	1	0.7
		Denmark	1	0.7
		Finland	1	0.7
		Ireland	1	0.7
		Italy	1	0.7
		Netherland	1	0.7
		Portuguese	1	0.7
		Slovakia	1	0.7
	Asia (n = 4)	South Korea	1	0.7
		China, Congo, Croatia, India, Turkey	1	0.7
		Japan	1	0.7
		Malaysia	1	0.7
	Oceania	Australia	5	3.5
	Africa	Kenna	1	0.7
		Not described	22	15.5
Study design	Experimental/Cohort study (n = 76)	RCT/experimental study	33	23.2
		Quasi experimental study (including one group)	18	12.7
		Observational/Case/Cohort study	25	17.6
	Developmental study(n = 58)	Measurement developmental study	27	19.0
		Scenario developmental study	16	11.3
		Program (Simulation) developmental study	15	10.6
	Others(n = 8)	Mixed methods	1	0.7
		Non categories	7	4.9
Typology of simulation	Standardized patient/participant(n = 10)	SP	9	6.3
		SP + peer to peer (role play)	1	0.7
	Computer-based training(n = 30)	Computer based simulation	2	0.7
		Computer based simulation (App)	1	0.7
		Computer based simulation (Haptic-enabled hand)	1	0.7
		Web based simulation	5	3.5
		Web based simulation + High-fidelity simulation	2	1.4
		Web based simulation + Video based simulation	1	0.7
		Video based simulation	17	20.2
		Audio simulation	1	0.7
	Advanced patient simulator(n = 92)	High-fidelity simulation	75	52.5
		High-fidelity simulation + SP	4	2.8
		High-fidelity/Mid-fidelity/Low-fidelity simulation/SP	1	0.7
		High-fidelity simulation + OSCE	1	0.7
		High-fidelity simulation + Video based simulation	1	0.7
		High-fidelity simulation + VR simulation + SP	1	0.7
		Low-fidelity simulation	3	2.1
		Low-fidelity simulation + OSCE	1	0.7
		Manikin based simulation	4	2.8
		Manikin based simulation + OSCEs	1	0.7
	Virtual reality	VR simulation	4	2.8
	OSCE	OSCEs	2	1.4
	Peer to Peer	peer to peer (role play)	3	2.1

Footnotes: App, application; OSCE, objective structured clinical examination; RCT, randomized controlled trial; SP, standardized patient; VR, virtual reality

#### Analysis of simulation scenario contents

A total of 98 studies were included in the analysis of the P-SBE scenario contents (Table 2). The most common target population of P-SBE was medical staff (n = 44, 44.9%), more specifically, there were 37 (37.8%) studies on medical students, medical residents, or medical fellows and seven (7.1%) studies on medical doctors or medical experts. Of the studies conducted on nursing staff, eight (8.2%) studies were conducted on nursing students, and three (3.1%) studies were conducted on registered nurses or experts. Four (4.1%) studies were conducted on children or students, and three (3.1%) studies were conducted on parents. The most common number of scenarios included in the analysis software was one (n = 49, 50.0%), followed by four (n = 13, 13.3%). The proficiency levels were competency (n = 29, 29.6%), proficient (n = 29, 29.6%), and expert (n = 10, 10.2%). Scenario contents included emergency intervention (n = 59, 60.2%), communication ability and decision-making (n = 19, 19.39%), and protection and safety (n = 17, 17.35%). Specific topics included pediatric rescue (n = 37, 37.8%), neonatal rescue (n = 11, 11.2%), and airway management (n = 8, 8.2%). Among the programs developed for children, two studies developed a simulation to enhance the decision-making ability of children with autism spectrum disorder (ASD) [15, 16], and programs developed for students targeted to train rescue competencies [17] and enhance decision-making ability in relation to cigarette smoking [18]. Seventy-two (73.5%) studies had self-developmental scenarios, and 23 (23.5%) had already been published. In terms of validity and reliability evaluation, 48 studies (49.0%) did not test validity, and 55 studies (56.1%) did not test reliability (Table 2). The most common type of validity tested was content validity (n = 10, 10.2%) and the most common type of reliability tested was inter-rater reliability (n = 10, 10.2%) (Table 3).

**Table 2:** Characteristics of Simulation program and scenarios (n = 98)

Characters	Categories	Subcategories	n	percent
Subjects	Medical member(n = 44)	Students or residents or fellows	37	37.8
		Medical doctors or medical experts	7	7.1
	Nursing member(n = 11)	Nursing students	8	8.2
		RN or Nurse practitioners	3	3.1
	Medical member + Nursing member		11	11.2
	Medical member + Nursing member + Others		13	13.3
	Parents		3	3.1
	Children or students		4	4.1
	Paramedics or lifeguards, respiratory therapist, etc.		12	12.2
Number of scenarios	1.00		49	50.0
	2.00		10	10.2
	3.00		12	12.2
	4.00		13	13.3
	5.00		6	6.1
	6.00		4	4.1
	7.00		2	2.0
	8.00		1	1.0
	9.00		1	1.0
Level of proficiency	Novice		9	9.2
	Advanced beginner		21	21.4
	Competent		29	29.6
	Proficient		29	29.6
	Expert		10	10.2
Contents of scenario	Emergency intervention (n = 59)	Pediatric rescue	37	37.8
		Neonatal rescue	11	11.2
		Airway management	8	8.2
		Respiratory support	3	3.1
	Protection & safety (n = 17)	Protection for child	6	6.1
		Skills for injection	3	3.1
		Medication error	1	1.0
		Pediatric disaster triage	1	1.0
		Trauma care	1	1.0
		Weight estimate	1	1.0
		Care for newborn, infant with symptoms	4	4.1
	Communication ability & Decision making (n = 19)	Communication skills	6	6.1
		Critical decision	13	13.3
		Decision making ability	2	2.0
		Health care skills(parent)	1	1.0
Self-developmental scenario	Yes		72	73.5
	Published already		23	23.5
	Not described		3	3.1
Number of doing validity and reliability for scenario	Verify the validity	Yes	30	30.6
		Published already	20	20.4
		Not described	48	49.0
	Verify the reliability	Yes	23	23.5
		Published already	20	20.4
		Not described	55	56.1

Footnotes: RN, registered nurse

**Table 3:** Specific analysis on the simulation program and scenario

No	Author, Year	Subject (n)	Competency	Type of simulation	Number of scenario	Level of proficiency	Scenario (contents)	Self-developmental scenario	Validity methodology	Reliability methodology
1	Abraham, 2016 [34]	MF(12), MR(12)	J	HFS	4	4	Critical decision :(a) diabetic ketoacidosis case (b) pyruvate dehydrogenase deficiency (c)pyridoxine-dependent epilepsy (d) supraventricular tachycardia with aberrancy	Y	Contents validity: expert	ND
2	Adeyinka, 2013 [35]	MS(30)	S	HFS	1	2	Airway management: Pediatric airway management	N	ND	ND
3	Adler, 2007 [36]	MR(54)	J	HFS	4	3	Critical decision: (a) apnea (ingestion), (b) asthma, (c) supraventricular tachycardia, (d) sepsis (oncology patient)	Y	Contents validity: expert	ICC
11	Appelbaum, 2019 [37]	MD(30), RN(30)	S	HFS	2	4	Medication error: (a) Prolonged status epilepticus(8-month-old, 8 kg child) (b)Presumed meningococcal sepsis (10-month-old, 9 kg child).	Y	Face validity	Interobserver reliability
17	Aye, 2014 [38]	MS(44)	A	SP	1	2	Communication ability: adolescents with various psychosocial issues	NS	ND	ND
26	Bigelow, 2000 [39]	Parent(7)	S	role-play (PtP)	1	1	Health care skills training: parents were provided with training in parent-child interaction skills and home safety and cleanliness.	Y	CVI	Interobserver Reliability
33	Brett-Fleegler, 2008 [40]	MR(25)	S	HFS	1	3	Pediatric rescue: A 14-year-old boy presents to the emergency department after a lake accident; witnessed to flounder and go under.	N	DA (by Brett-Fleegler & Kleinman)	DA
34	Brown, 2018 [41]	PRN(30)	A	HFS	2	3	Heart surgery ability: (a)postoperative hypoplastic left heart syndrome (HLHS) patient following the Norwood procedure with Blalock-Taussig (BT) shunt and pulmonary over circulation (b) a patient with pulmonary hypertensive crisis following atrioventricular canal repair.	Y	Face validity	ND
35	Brubacher, 2015 [42]	Teacher(36)	A	VRS + Mock interview	1	2	Communication ability: open-ended questions: Unreal Interviewing. In the exercise, participants choose the best question (out of four options) to ask a child avatar. The avatar responds to the question on the basis of research on children’s cognitive development.	Y	ND	ND
41	Burns, 2013 [43]	MR(28)	J	HFS	7	3	Critical decision: (a)wrist pain, (b)acute chest syndrome, (c)acute splenic sequestration crisis, (d)asthma exacerbation, (e)ceftriaxone-induced hemolytic anemia, (f)posterior reversible encephalopathy syndrome, (g)Vaso-occlusive pain crisis.	Y	Content validity: expert	ND
42	Byars, 2013 [44]	Paramedics(38)	S	HFS	1	2	Airway management: the tongue swelling was set at the most difficult setting with a large protruding tongue	Y	ND	ND
48	Chitkara, 2013 [45]	MR(22),MF(7), MD(9),hospitalists(7),NP(7),RN(10)	A	HFS	3	5	Respiratory support: (a) a vigorous term infant (39 wks. gestation) with spontaneous respiratory effort and an initial HR set at 130 BPM. In this scenario the appropriate intervention was to warm, dry and stimulate (W/D/S) (b) a non-vigorous, post-term infant (41 + weeks gestation) with minimal respiratory effort and an initial HR set at 90 BPM. W/D/S followed by PPV was the proper response to this scenario (c) a non-vigorous, apneic, term infant (40 weeks) born after acute blood loss due to placental abruption with initial HR set at 50 BPM.	Y	ND	ND
49	Chiu, 2014 [46]	49(2010year),306(2011year)	J	HFS	9	3	Critical decision: (a) asthma (b)congestive heart failure (CHF) (c) supraventricular tachycardia (SVT), 2) three pediatric scenarios, or 3) three obstetric scenarios.	N	DA (by UW)	DA
50	Cicero, 2014 [47]	ME(8)	J	HFS, MFS, LFS, SP	10	5	Pediatric disaster triage: a multiple-family house fire, a school shooting, and a school bus rollover. Each 10-victim simulation had similar injury severity and inclusion of 10 total infant, child, adolescent, and adult disaster victims.	N	DA (by Ballow et al.) + modified delphi	DA
55	Cordero, 2013 [48]	11team (MR(2), intern(1)/ team)	S	HFS	1	4	Neonatal rescue: a 37-week GA infant born by cesarean delivery to a preeclamptic woman with placental abruption. APGAR scores were 1 at 1 and5.	N	DA(SimNewB NRP 2010)	DA
56	Cordova, 2018 [49]	MD + RN+ midwives(80)	S	MBS + OSCEs	1	5	Neonatal rescue: HBB: not breathing infant- it appropriately in a simulated resuscitation scenario.(NeoNatalie)	N	DA(by the American Academy of PD	DA
58	Costa, 2019 [50]	NS(39)	S	HFS	1	2	Skill of injection: the administration of vaccines in the vastus lateralis muscle of the thigh in children was developed	Y	ND	ND
60	da Costa Brasil, 2018 [51]	NS(47)	J	HFS	4	2	Critical decision: (a) a presentation of pre-eclampsia, (b)newborn resuscitation, (c)pneumonia in an infant, (d)trauma-induced placental abruption, (e)violence against women and family planning consultation.	NS	ND	ND
66	do Nascimento Targino, 2021 [52]	NS(30)	S	MBS	1	2	Neonatal rescue: the maneuver to disengage the lactant subsequently in the event of cardiorespiratory arrest (PCR) and perform the cardiopulmonary resuscitation (RCP) in infants.	Y	ND	ND
69	Donoghue, 2010 [53]	MR(20)	S	HFS	4	3	Pediatric rescue: (a)a systolic, (b)dysrhythmia, (c)respiratory, (d) shock scenario (PALS)	N	DA (PALS)	coefficients/standardized coefficient of inter-rater
71	Donoghue, 2011 [54]	Team leader ( MR,NP)	S	HFS, LFS	2	4	Pediatric rescue: (a) hypovolemic shock, (b) ventricular fibrillation-return of spontaneous circulation	N	DA(PALS)	DA
73	Dorsey, 1996 [55]	MS(40)	K	CS	1	2	Protection for child: A case involving the sexual abuse of a 6-year-old girl(the child presents with headaches coinciding with her mother’s boyfriend moving into their household.)	Y	ND	ND
75	Edler, 2010 [56]	ANEstudents(48)		LFS	5	2	Airway management: The portable simulation training and assessment program (Pediatric Anesthesia in-Situ Simulation)	NS	Delphi	ND
76	Edwards, 2018 [57]	parents(15)	S	VBS	1	1	Skill of injection: 0.1 mg EAI use education. Anaphylaxis in infants and young children, epinephrine injection	Y	ND	ND
79	Everett, 2019 [58]	ANE students (154)	A	HFS	6	2	Airway management: (a) anaphylaxis, (b) equipment failure, (c) hypovolemia, (d) local anesthetic toxicity, (e)laryngospasm, retained throat pack, (f) malignant hyperthermia).	Y	ND	ICC, GRS
88	Finan, 2012 [59]	neonatal MF(16)	J	HFS, LBS	2	4	Neonatal rescue: (a) a term infant, delivered through meconium-stained liquor and in poor condition at birth. tension pneumothorax requiring thoracocentesis. Failure to recognize the air leak resulted in further decompensation and cardiac arrest. (b) a term infant, on the postpartum ward, who developed supraventricular tachycardia. initially hemodynamically stable, trainees were expected to recognize the supraventricular tachycardia and institute appropriate vagal maneuvers and medical therapy.	Y	ND	ND
96	Geis, 2018 [60]	PD(18)	J	HFS	4	5	Critical decision: one “garden path” simulation, two scenarios of compensated sepsis, and two scenarios of uncompensated septic shock	Y	ND	ND
97	Gerard, 2018 [61]	MS(60)	J	GS	7	2	Critical decision: The game features seven scenarios depicting critical pediatric medical diseases including (a) anaphylaxis, (b) bronchiolitis, (c)diabetic ketoacidosis, (d)respiratory failure, (e)seizure, (f)septic shock, and (g)supraventricular tachycardia. Patients range from the ages of 3 weeks to 10 years.	Y	NS(AAP)	Cronbach’ a
100	Grant, 2012 [62]	Physician educator(8)	S	VBS	1	4	Pediatric rescue: require airway, breathing, and circulation assessment along with recognition and treatment of cardiac arrhythmias and shock.	N	ND	ND
102	Hall, 2015 [63]	PD or MD(50)	K,S	HFS, SP	1	5	Protection for child: Child abuse victim-a physically abused neglected child, the unexpected death of an infant in a difficult social context and the possible sexual exploitation of a young teenager.	Y	ND	ND
104	Hasselager, 2018 [64]	lifeguards(33)	S	VBS	1	3	Airway management: an infant with sudden foreign body airway obstruction with rapid deterioration into unconsciousness.	Y	ND	ND
106	Heimberg, 2014 [65]	MD(47),RN(49)	J	HFS	1	4	Critical decision: A standardized septic shock scenario of a 6-month old boy admitted to hospital	Y	ND	ND
107	Herzberg, 2019 [66]	EMS(259)	S	HFS	4	2	Pediatric rescue: (a) cardiac arrest in newborn (b) cardiac arrest in child(c) nonaccidental trauma (d) accidental trauma from pedestrian motor vehicle collision	N	DA(PALS, NAT)	DA
108	Hodgkinson, 2019 [67]	RN(10), MD(14)	S	SP	1	4	Communication skill: using a professional actor to practice discussing difficult topics, including breaking bad news, discussing post mortem and safeguarding concerns.	Y	ND	ND
109	Hossino, 2018 [68]	MR(26)	S	HFS	1	3	Neonatal rescue: leader, airway, chest compressions, and umbilical venous line placement.	Y	ND	ND
111	House, 2012 [69]	EM MR(49)	S	HFS	1	3	Pediatric rescue: a 7-month-old infant in respiratory failure	Y	Delphi	ND
113	House, 2016 [70]	Parents(99)	K	VBS	1	1	Protection for child: ATV(all-terrain vehicle) video animation	Y	ND	ND
114	Hoyle, 2020 [71]	EMS(109)	S	HFS	4	2	Airway management & Infant rescue: (a) an infant with a seizure who was also hypoglycemic, (b) an 18-month-old with a partial thickness burn, (c) a 5-year-old with anaphylactic shock, and (d) an infant in cardiac arrest	Y	ND	ND
115	Hunt, 2007 [72]	EM RN(18)	S	HFS	1	3	Trauma care: Trauma(a 3 year old, had fallen off of a tall slide)	Y	ND	ND
118	Jabbour, 2012 [73]	MS(3), otolaryngology MR(17),pediatric otolaryngology faculty(3)	S	VBS	1	4	Airway management: a 6-month-old who has just arrived to the operating room because of concern for an airway foreign body	Y	ND	ND
120	John, 2019 [74]	PMR(6)	S	HFS	1	3	Pediatric rescue: pediatric emergencies scenario	N	ND	ND
123	Kalaniti, 2019 [75]	Pediatric trainees(22)	S	HFS	1	3	Neonatal rescue: neonatal resuscitation scenario	N	ND	ND
124	Kane, 2019 [76]	Experienced resuscitators(102)	S	HFS	1	4	Neonatal rescue: neonatal rescue-resuscitation scenarios	Y	ND	ND
126	Katznelson, 2018 [77]	ED employees(150)	S	HFS	1	4	Neonatal rescue:- Pediatric Resuscitation	Y	ND	ND
127	Keidan, 2008 [78]	PD(30),ANE MR (10)	S	HFS	1	4	Respiratory support: apnea in a 6-year-old patient who received sedation for resetting of a fractured leg.	Y	ND	ND
129	Khan, 2020 [79]	MR(12)	S	LFS	1	4	Respiratory support: tracheostomy and ventilator-dependent patient, tracheostomy dependent patient	Y	ND	ND
130	Khorram-Manesh, 2018 [17]	Students(25)	K	HFS	1	1	Pediatric rescue: Emergency management and preparedness training for youth[EMPTY].	Y	ND	ND
131	Kim, 2014 [80]	NS(147)	J	HFS	1	2	Critical decision: high fever & seizure :15 month baby were admitted via emergency room [ER].	Y	Content validity	ND
132	King, 2016 [81]	Clinician(4), Researchers(3) Pediatric rehabilitation manager(1), Senior directors(2)	A	VBS	2	4	Communication ability: (a) 3 year-old girl with a speech and language disorder. (b) 8-year-old boy with Duchenne muscular dystrophy.	Y	Focus group interview	ND
133	Kioko, 2010 [82]	PMR(6), PICURN(2)	S	HFS	5	3	Pediatric rescue: the resuscitation of critically-ill pediatric patients scenarios(5 cases)	Y	ND	ND
135	Kothari, 2021 [83]	EMS(313)	S	HFS	3	4	Pediatric rescue: (a) 15-month-old with septic shock and seizure, (b) 1-month-old with hypoglycemia, hypovolemic shock, (c)4-year-old clonidine ingestion	Y	construct validity	IRR
137	Kurosawa, 2014 [84]	RN + RRT(40)	S	HFS	2	5	Pediatric rescue: (a)hypovolemic shock + ventricular fibrillation, (b)asthma + distributive shock	N	DA(PALS)	DA
139	Lacour, 2021 [85]	Paramedics(150)	S	HFS	1	1	Pediatric rescue: highly realistic pediatric Out-of-hospital cardiac arrest CPR scenario	Y	ND	ND
140	LaFond, 2015 [86]	RN(4)	A	HFS	4	3	Protection of child(pain): (a) child first postoperative day abdominal surgery, smiling (b) child first postoperative day abdominal surgery, grimacing (c) child with sickle cell vaso-occlusive crisis, smiling(d) child with sickle cell vaso-occlusive crisis, grimacing	Y	Face validity, Convergent validity	Interview
141	Lammers, 2009 [87]	EMS(12)	S	MBS	3	4	Pediatric rescue:(a) arrest (b) asthma (c)sepsis	Y	ND	ND
142	Lammers, 2022 [88]	EMS(147)	S	HFS, LFS	3	4	Pediatric rescue: (a)arrest (b) asthma) (c)sepsis/seizure	Y	ND	ND
146	Larsen, 2018 [18]	Students(81)	J	AS	1	1	Critical decision: someone offers student a cigarette’ and their behavior	Y	face validity	ND
147	LeBlanc, 2012 [89]	Child protection workers(96)	S	SP	2	3	Protection of child: (a) an interview with a mother (Ms. Smith) of an infant following a report by the child’s daycare provider that welts had been observed on the child. (b) an interview with the mother of a latency-aged child following the report by a school that the child had disclosed physical abuse.	Y	Content validity, Focus group interview	ND
148	Lee, 2012 [90]	MR(27)	S	HFS	4	4	Neonatal rescue: (a) Health term neonate, (b) neonate with bradycardia, (c) Neonate with bradycardia, (d) Neonate with apnea and bradycardia	Y	ND	ND
150	Lemke, 2019 [91]	PMF(21), RN(8)	S	HFS	3	4	Pediatric rescue: (a)unstable SVT and high output heart failure (b) upper airway obstruction and asystolic arrest(c) lower respiratory obstruction and ventricular fibrillation	N	DA (by American Heart Association)	ICC
152	Levy, 2014 [92]	PMR(24)	S	HFS	5	3	Pediatric rescue: (a)pulseless nonshockable arrest, (b)pulseless shockable arrest, (c)dysrhythmia, (d)respiratory arrest, (e)shock	Y	ND	ND
154	Levy, 2012 [93]	PMR(24)	S	VBS	6	3	Pediatric rescue:(a) pulseless non-shockable arrest: asystole or pulseless electrical activity (b) Pulseless shockable arrest: ventricular tachycardia or ventricular fibrillation Tachycardia (c)Tachycardia with Poor Perfusion (Supraventricular Tachycardia (SVT) or Ventricular Tachycardia With Pulse) (d) respiratory arrest, apnea or post-seizure (e) shock: hypovolemic shock or septic shock; (f)dysrhythmia: supraventricular tachycardia or stable ventricular tachycardia	Y	ND	ND
159	Marlow, 2013 [94]	MS + RN(57)	S	CBS	1	3	Calculation: weight estimation	Y	ND	ND
163	McBride, 2011 [95]	MR(29)	J	HFS	1	3	Pediatric rescue: (a) ventricular tachycardia (b) pulseless electrical activity (c) tension pneumothorax at delivery (d) non-accidental trauma (e) status epilepticus (f) bronchiolitis (g) traumatic brain injury (h) ventricular septal defect (i) asthma (j) primary apnea at delivery (k) critical coarctation (l) 28-weeker delivery (m) narcotic overdose (n) diabetic ketoacidosis (o) abdominal trauma (p) septic shock (q) supraventricular tachycardia (r) croup (s) meconium aspiration delivery (t) acute gastroenteritis	Y	Content validity, Focus group interview	ND
169	Mema, 2016 [96]	PMF(17)	A	OSCE	8	3	Pediatric rescue: (a)arrhythmia, (b)chest tube insertion, (c)breaking bad news, (d)brain death, (e)transport call, (f)tracheostomy, (g)cardiac tamponade, (h)asthma	Y	face validity	ICC
177	Nadkarni, 2018 [97]	Physicians(2),RN(3–5), NA(2–3)	A	VBS	4	4	Pediatric rescue: (a) child cardiac arrest (drowning), (b) infant respiratory arrest (foreign body), (c) infant seizure (hypoglycemia), and (d) infant sepsis (bacteremia).	Y	ND	ND
187	Neira, 2013 [98]	ANE MR(50)	S	VBS	2	4	Airway management: (a) pediatric anesthesia scenarios (laryngospasm, and hyperkalemia), (b)laryngospasm scenario	Y	ND	ND
190	Padhya, 2021 [99]	PICU MD+ RN + MR(18)	A	WBS, VBS	3	4	Pediatric rescue: (a) Hypotension due to urosepsis; (b) Respiratory distress in the setting of community acquired pneumonia; (c) Status epilepticus	Y	ND	ND
192	Ponce de Leon, 2018 [100]	NS(10)	S	HFS	2	2	Protection for child: (a) adolescents’ use of licit and illicit drugs and sexual abuse of a minor; (b) Early sexual initiation, pregnancy, and abortion among adolescents.	Y	CVI	ND
202	Rovamo, 2011 [101]	Consultant neonatologists(6), PMD(11), ANE MD(11)	S	HFS	1	5	Neonatal rescue: a standard scenario with a newborn infant with severe asphyxia	N	DA(standard scenario :first Finnish national neonatalresuscitation)	DA
203	Rowe, 2012 [102]	Health workers in the same 55 health facilities.	J	SP	6	3	Critical decision: the child had fever, diarrhea, and one episode of vomiting with no signs of severity or other illnesses.(6 cases with SC) 6-59 m.	Y	CO bias, compared to an SC gold standard, CO methodology	sensitivity analyses: simple CO – SC estimate of CO bias
205	Russo-Ponsaran, 2018 [15]	Children with ASD(21), control children(29)	S	VRS	5	1	Decision making: Virtual Environment for SIP (VESIP(TM) ), a simulation-based assessment that immerses children in social decision-making scenarios within a school environment- two simulated school days, 5scenarios/d)(friendly helper, respondent select multiple choice, slider option)	Y	literature review, usability, feasibility-initial validity는 feasibility testing	Internal Consistency Reliability
207	Sadideen, 2014 [103]	MD(novice and expert)(12)	S	HFS + VRS + SP	1	4	Pediatric rescue: “The Burns Suite(TBS)” burns scenario:pediatric burn resuscitation scenario -ATLS(3d), EMSB(1d)	Y	face validity, ccontent validity:expert	Cronbach’s α
208	Sadideen, 2016 [104]	Participants (MD, RN, and NA)	S	HFS + SP	1	4	Pediatric rescue: A realistic pediatric burn resuscitation scenario	Y	content validity	Cronbach’s α
209	Sagalowsky, 2018 [105]	PMR(33)	K	HFS	1	3	Pediatric rescue: Simbaby scenario	N	DA(Simbaby:Laerdal)	IRR
210	Scalon da Costa, 2019 [106]	NS(39)	S	LFS + OSCE	1	2	Skill of injection: administration of vaccines in the vastus lateralis muscle of the thigh in children as a proposal of intervention,	Y	content validity: expert (Ministry of Health)	Cronbach’s α
212	Schmutz, 2014 [107]	Expert(15)	S	VBS	1	5	Pediatric rescue: infant septic shock. developing checklists to rate clinical performance is essential for ensuring their quality	Y	Delphi-Internal consistency and validity	IRR
213	Schmutz, 2015 [108]	Training sessions(50)	S	HFS	3	3	Pediatric rescue: (a)cardiopulmonary arrest, (b)dyspnea with oxygen desaturation after intubation, and (c)respiratory syncytial virus (RSV)	Y	Construct Content	IRR
214	Sepúveda Oviedo, 2022 [109]	MR(ND)	A	HFS	1	3	Neonatal care: even physiological scenarios: two of them representing a healthy infant (newborn and 6-months old) and five representing newborns affected by different heart diseases.	Y	ND	ND
216	Seto, 2017 [110]	MD(31),RN(39)	S	HFS + OSCE	2	5	Neonatal rescue: Helping Babies Breathe (HBB) is a simulation-based neonatal resuscitation curriculum -n A is a routine newborn care scenario, whereas OSCE B is a more complex neonatal resuscitation scenario that requires learners to perform BMV	N	DA(HBB)	DA
218	Shin, 2014 [111]	NS(250)	S	VBS	1	2	Pediatric care: The febrile infant care	Y	Content Validity. Convergent Validity. Construct Validity	Cronbach’s α
220	Siebert, 2022 [112]	RN(50), MR(51)	S	HFS	3	3	Pediatric rescue: cardiopulmonary scenarios : (a)defibrillation, (b)cardioversion, and (c)transcutaneous pacing)	N	DA(PALS)	DA
221	Sigalet, 2012 [113]	MS(1) + NS (3–4), +RTS(1), total = 196	A	HFS	1	2	Pediatric rescue: 3-hour IPE curriculum module that focused on 2 simulation-based team training scenarios in emergency and intensive care unit settings.	Y	ND	ND
224	Smith, 2019 [114]	MR(ND)	S	CBS	4	3	Critical decision: (a)Lower respiratory tact infection (LRTI), (b)Lower airway obstruction (LAO), (c)Hypovolemic shock from severe dehydration (HSSD), (d)LRTI with distributive shock from sepsis (LRTI + DSS)	Y	Delphi	ICC
228	Teis, R, 2017 [115]	MD(25), RN(25), or NM(25), three intervention groups (n = 24)	S	HFS	6	5	Critical decision: Crisis Resource Management (CRM) skills including communication, leadership, knowledge of environment, teamwork, anticipation and planning, attention allocation, workload distribution and use of cognitive aids are of core importance to the practice of emergency medicine	Y	DA(Crisis Resource Management (CRM) )	DA
229	Tobler, K, 2014 [116]	MR(39)	A	SP	3	3	Communication skill: (a)Near drowning of a 5-month-old that progresses to brain death (b) Inflicted brain injury in a 4-month-old with an angry grandparent present for the second encounter (c)Traumatic brain injury of a 1-year-old in the context of parental discord.	Y	ND	ND
230	Tofil, N. M, 2017 [117]	Team leaders(127), Team members(254)	A	HFS	1	4	Pediatric rescue: Simulated sepsis scenario(12-minute pediatric sepsis simulation scenario.)	N	DA(The National Aeronautics and Space Administration)	DA
232	Traynor, 2021 [118]	Orientees(48) ICU nurse preceptors(11)	S	HFS	3	4	Pediatric rescue: (a) respiratory failure: a patient with acute respiratory failure (b) sepsis: a patient experiencing sepsis; and (c) neurological failure: a patient with hydrocephalus and an external ventricular device	Y	Content validity: expert	ND
233	Tsai, T. C, 2003 [119]	PMR(18)	S	HFS	5	4	Pediatric rescue: (a) severe asthma with pneumothorax, and (b) diarrhea with severe dehydration. The post-test cases were: (c) car crash complicated with pneumothorax and chest contusion, and (d) insulin-dependent diabetes mellitus (f)diabetic ketoacidosis.	Y	construct validity	IRR
235	Tyler, 2021 [120]	Social workers undergraduate students(37)	S	SP	1	2	Communication ability: a simulation scenario with either a parent and bisexual child or a parent and transgender child.	Y	ND	ND
238	Ventre, K. M., 2009 [121]	Participate(ND)	S	CBS(VR)	4	2	Pediatric rescue: (a)supraventricular tachycardia, (b)pulseless electrical activity, (c)ventricular fibrillation, (d) bradycardia)	N	DA(PALS)	DA
240	Wallace, 2010 [16]	Children with ASD(10)/typically developing (TD)(14)	S	VR simulation	5	1	Decision making: the present study was carried out to explore how young people with ASD experience and respond to an immersive virtual environment in which highly realistic representations	Y	Focus group interview	ND
242	Walton, J. L, 2018 [122]	RRT(17)	S	HFS	3	2	Pediatric rescue: (a) a 2-month-old male infant with respiratory distress requiring pressure control ventilation,(b) a 10-y-old male with status epileptic requiring volume control ventilation, (c) a 16-y-old female with severe cog native deficiency requiring noninvasive ventilation.	Y	ND	ND
243	Watkins, S. C., 2021 [123]	Certified nurse anesthetist or ANE MR	S	HFS	4	4	Pediatric rescue: (1) hyperkalemia that progresses to ventricular fibrillation, (2) supraventricular tachycardia (SVT) that progresses to pulseless ventricular tachycardia, (3) anaphylaxis that progresses to pulseless electrical activity, or (4) local anesthetic toxicity that progresses to asystole	N	DA (http://links.lww.com/SIH/)	DA
245	Watkins, S. C, 2017 [124]	Novices(4), Experts(2)	S	VBS	3	3	Pediatric rescue: (a)hypoxemia (hypoxia), (b)ventricular fibrillation, (c)supraventricular tachycardia (SVT).	Y	ND	IRR
248	Whalen, A. M., 2018 [125]	MS, MR, MF	S	HFS	1	3	Pediatric rescue: neonatal and pediatric BMV skills.	N	Delphi process Criterion validity	IRR
249	Whalen, A. M., 2022 [126]	Expertise or MS(58)	S	HFS	1	4	Pediatric rescue: pBMV Simulation Setting and Scenario	N	DA	DA
252	Naoko NAMBA, 2021 [127]	NS(ND)	S	VBS	1	1	Neonatal care: Newborn early care	Y	ND	ND

Footnotes: AAP, American academy of pediatrics; ANE, anesthesiology; AS, audio simulation; ASD, Autism Spectrum Disorders; CBS, computer based simulation; CO, conspicuous observation; CVI, content validity index; DA, development already; ED, emergency department; EM, emergency; EMS, emergency medical technician; GA, gestational age; GS, game simulation; GRS, global rating scale; HBB, helping babies breathe; HFS, high fidelity simulation; ICC, intraclass correlation coefficient; IRR, intra-rater reliability; LFS, Low fidelity simulation; PRN, pediatric registered nurse; RN, registered nurse; TRACS, tool for resuscitation assessment using computerized simulation; MBS, manikin-based simulation; MD, medical doctor; ME, medical expert; MF, medical fellow; MFS, middle fidelity simulation; MR, medical resident; MS, medical student; NA, nursing assistant; NAT, non-accidental trauma; ND, not described; NM, nursing manager; NP, nurse practitioner; NS, nursing student; OSCE, objective structured clinical examination; PALS, pediatric advanced life support; PD, pediatric; PD, pediatric doctor; PICU, pediatric intensive care unit; PMF, pediatric medical fellow; PMR, pediatric medical resident; RT, respiratory therapists; RRT, registered respiratory therapist; RTS, respiratory therapy student; SC, simulated client; SP, standardized patient; VBS, video based simulation; VRS, virtual reality simulation

#### Outcome variables of simulation program

Of the studies that used one or more outcome variables, most (n = 65, 66.3%) used the skill category as the outcome variable, namely skills, performance, assessment, and communication skills. Twenty-six (26.5%) studies used the attitude category as the outcome variable, namely attitude, confidence, satisfaction, and stress. Seventeen (17.3%) studies have examined this knowledge. Fifty-six studies (57.1%) used one outcome variable and 31 (31.6%) used more than one outcome variable. Sixty-six (67.3%) studies did not test validity, while 50 (51.0%) did not test reliability (Tables 4 and 5).

**Table 4:** Outcome Variables of Scenarios (n = 98)

Characters	Categories	Subcategories	n	percent
Type of Variables	Knowledge		17	17.3
	Competencies (n = 65)	Skills	28	28.6
		Performance	24	24.5
		Assessment	9	9.2
		Communication skills	4	4.1
	Attitude (n = 26)	Attitude	4	4.1
		Confidence	12	12.2
		Satisfaction	6	6.1
		Stress	4	4.1
Number of outcome variables	1		56	57.1
	Above 1		31	31.6
	Not described		11	11.2
Number of doing validity and reliability for outcome variables	Verify the validity	Yes	32	32.7
		Not described	66	67.3
	Verify the reliability	Yes	48	49.0
		Not described	50	51.0

**Table 5:** Specific analysis on the simulation program and scenario

No	Author, year	Categories of outcomes	Variables	Scales	Verify validity	Verify reliability
1	Abraham, 2016 [34]	Skills	Critical-action score (CAS)	Critical-action checklist	N	N
2	Adeyinka, 2013 [35]	Skills	Psychomotor skills required for pediatric intubation	Using a validated scoring tool adopted from Kovacs et al.	Y	Y
3	Adler, 2007 [36]	ND	ND	ND	N	N
11	Appelbaum, 2019 [37]	Skills-number of errors	Medication error	Data management and analysis	Y	N
17	Aye, 2014 [38]	Knowledge, Confidence, Communication skill, Effectiveness	Knowledge, clinical confidence, communication skills, and effectiveness of simulated clinical teaching.	Self- development	Y	N
26	Bigelow, 2000 [39]	Knowledge/ Assessment	Parent knowledge, simulated performance in identifying symptoms, treating illnesses and injuries, and seeking appropriate treatment	Self- development	N	N
33	Brett-Fleegler, 2008 [40]	Competency	Pediatric resuscitation competency	Pediatric Resuscitation Competency Tool	N	Y
34	Brown, 2018 [41]	Knowledge/ Confidence/ Satisfaction	Knowledge, confidence, satisfaction	Student Satisfaction and Self-Confidence in Learning” tool)	N	N
35	Brubacher, 2015 [42]	Communication skill	Open-ended, specific, leading, and minimal encourager	Coding & number	N	Y
41	Burns, 2013 [43]	Performance	Performance, preparedness, usefulness	Likert 5 scale	N	N
42	Byars, 2013 [44]	Skills	Ventilation time	Seconds	N	N
48	Chitkara, 2013 [45]	Skills	Heart Rate check error	Video tape review	N	N
49	Chiu, 2014 [46]	Performance/Assessment Communicational skills	Performance Assessment Tools for Interprofessional Communication and Teamwork (PACT)	PACT	Y	Y
50	Cicero, 2014 [47]	Assessment	Pediatric disaster triage (PDT) performance	Pediatric disaster triage (PDT) checklist	N	N
55	Cordero, 2013 [48]	Skills,/ Performance	Procedural Skills: Technical Aspects/Procedural Skills: Timeliness/Team Behavior Scores/Acceptable Performance Scores	Ventilator apply time/chest compression time/adequate (each 4point)	Y	Y
56	Cordova, 2018 [49]	Knowledge/ skills	Knowledge and skill	Previously validated OSCEs	Y	Y
58	Costa, 2019 [50]	Knowledge/ Performance	Knowledge and performance	OSCE checklist	Y	Y
60	da Costa Brasil, 2018 [51]	Satisfaction, Confidence	Student Satisfaction and Self-Confidence in Learning Scale	The Student Satisfaction and Self-Confidence in Learning Scale	Y	Y
66	do Nascimento Targino, 2021 [52]	Knowledge	Compare the proportions of the right/wrong answers before and after training	ND	N	N
69	Donoghue, 2010 [53]	Performance	Clinical performance	Clinical Performance Tool	Y	Y
71	Donoghue, 2011 [54]	Performance	Clinical performance tool (CPT)-(clinical, behavioral, and cognitive knowledge).	0–2-point checklist (pulseless arrest algorithm of the PALS)	Y	Y
73	Dorsey, 1996 [55]	Attitude	Attitude, opinion about sexual abuse in childhood	Likert scale	N	N
75	Edler, 2010 [56]	Satisfaction	Simulation satisfaction	ND	N	N
76	Edwards, 2018 [57]	Knowledge/ Confidence	EAI ease of use, confidence, knowledge. IFU task error	Information and Instructions for Use(IFU)	N	N
79	Everett, 2019 [58]	ND	ND	ND	N	N
88	Finan, 2012 [59]	Performance/ Stress	Clinical performance, objective, subjective stress	Team performance scoring tools, subjective stress, solitary cortisol	N	N
96	Geis, 2018 [60]	Assessment/ Performance	Recognizing sepsis, physician Performance	The Situation Awareness Global Assessment Technique	Y	Y
97	Gerard, 2018 [61]	Knowledge/ Scenario score/ Satisfaction	Knowledge, simulation scenario score, game-based simulation	Self-development	N	Y
100	Grant, 2012 [62]	Leadership/Communication skill/Knowledge/Performance	leadership and communication skill, knowledge, clinical skill	LCS, KCS	Y	Y
102	Hall, 2015 [63]	Knowledge /Self-confidence	Knowledge and self-confidence	ND	N	N
104	Hasselager, 2018 [64]	Performance	Foreign body airway obstruction management skills	Pass/Fail Likert 5 scale	Y	Y
106	Heimberg, 2014 [65]	Knowledge	Evaluating adherence to sepsis guidelines	ND	N	Y
107	Herzberg, 2019 [66]	Teamwork scale	Teamwork	Clinical Teamwork Scale(0–10)	N	N
108	Hodgkinson, 2019 [67]	Knowledge/Confidence	Knowledge and confidence	Scale of 1, not at all confident, to 10, very confident	N	N
109	Hossino, 2018 [68]	Confidence	Confidence	5 point Likert scale	Y	N
111	House, 2012 [69]	Knowledge/ Skills	Pediatric rapid sequence intubation and knowledge	Objective Structured Assessment of Technical Skills (OSATS)	N	N
113	House, 2016 [70]	Attitudes/Beliefs/Perceived risk	Attitudes, beliefs, perceived risk associated with child and adult ATV use.	ND	Y	Y
114	Hoyle, 2020 [71]	Performance	Dose error	Directly observed all simulations in the simulation space and graded performance on a standardized scoring sheet	N	N
115	Hunt, 2007 [72]	Performance	Pediatric trauma performance	Likert 5 scale	N	Y
118	Jabbour, 2012 [73]	Skills	Technical skill	Objective measures list, OSATS, GRTS	Y	Y
120	John, 2019 [74]	Confidence	Confidence	10 point likert scale	N	N
123	Kalaniti, 2019 [75]	Stress	Anxiety/stress	Cortisol and self-report stress questionnaire	N	N
124	Kane, 2019 [76]	Skills	Neonatal Resuscitation skill	7th edition of the Neonatal Resuscitation guidelines.	N	Y
126	Katznelson, 2018 [77]	Skills	Pediatric resuscitation skill	Pediatric Advanced Life Support and Advanced Cardiac Life Support guidelines	N	N
127	Keidan, 2008 [78]	Performance’s time	Bag-mask ventilation time	PaCo2	N	N
129	Khan, 2020 [79]	Performance	Performance to apply the ventilation	0–2 scale, total 10 point	N	Y
130	Khorram-Manesh, 2018 [17]	Knowledge	Knowledge	0 (dissatisfaction) to 10 (complete satisfaction)	N	N
131	Kim, 2014 [80]	Satisfaction	Simulation experience satisfaction	Satisfaction of Simulations Experience Scale	Y	Y
132	King, 2016 [81]	Complexity	Simulation complexity	Complexity rating scale	N	N
133	Kioko, 2010 [82]	Management skill	Weight-based drug dosages in the management	Crisis resource management (CRM) tool, Broselow-Luten Pediatric System	N	N
135	Kothari, 2021 [83]	Performance	Simulation performance	SimulationTeam Assessment Tool	N	N
137	Kurosawa, 2014 [84]	Skill/Behavioral performance	Skill performance, Behavioral performance	Clinical Performance Tool (CPT).Behavioral Assessment Tool (BAT),	N	Y
139	Lacour, 2021 [85]	Stress	Perceived stress	Spielberger’s psychometric State-Trait Anxiety Inventory (STAI) questionnaire, VAS	N	N
140	LaFond, 2015 [86]	Pain assess	Pain Beliefs and Practices Questionnaire	PBPQ	N	Y
141	Lammers, 2009 [87]	Skills	Pediatric resuscitation skill	Clinical Assessment Module Questionnaire	N	Y
142	Lammers, 2022 [88]	Skills	Pediatric resuscitation skill	Clinical Assessment Module Questionnaire	N	Y
146	Larsen, 2018 [18]	Behavior willingness/Expectancies	Smoking behavior, Behavioral willingness on S-SIDE, Self-reported willingness to smoke, Smoking Expectancies	Fagerströ Test, 7pint likert, 7pint likert, The short Smoking Consequences Questionnaire (S-SCQ)	N	Y
147	LeBlanc, 2012 [89]	Stress/ Assessment	Stress, Risk assessment	subjective measure (STAI) and cotisol, Ontario Risk Assessment Measure	N	Y
148	Lee, 2012 [90]	Confidence	Confidence	developed by the investigators (4 point likert scale)	N	N
150	Lemke, 2019 [91]	Assessment/Satisfaction	Rapid cycle deliberate practice, satisfaction	Simulation Team Assessment Tool	N	N
152	Levy, 2014 [92]	Performance	Scenario performance	CPT(Clinical performance tool)	N	Y
154	Levy, 2012 [93]	Error performance/ time	Resuscitation delay and error	PALS	Y	Y
159	Marlow, 2013 [94]	Assessment	Accuracy of weight estimation	ND	N	N
163	McBride, 2011 [95]	Confidence	Scenario confidence	Checklist and global rating scale	N	N
169	Mema, 2016 [96]	Performance	Scenario performance	ND	Y	N
177	Nadkarni, 2018 [97]	Performance	Resuscitation leader performance	Concise Assessment of Leader Management (CALM)	Y	-
187	Neira, 2013 [98]	Assessment	Generic Integrated Objective Structured Assessment Tool (GIOSAT)	GIOSAT	Y	Y
190	Padhya, 2021 [99]	Performance	Scenario performance	Clinical performance assessment	N	N
192	Ponce de Leon, 2018 [100]	Assessment	Simulation assessment	Expert characterization questionnaire, high-fidelity scenario validation tool	Y	N
202	Rovamo, 2011 [101]	Technical skills	A case-based checklist of technical skills that comprised 30 items was compiled using items from previous studies.	Technical skills.	Y	Y
203	Rowe, 2012 [102]	ND	NS	ND	N	N
205	Russo-Ponsaran, 2018 [15]	Preference/ Assessment	Solution preference/Problem identification/Intent attribution./Goal preference	VE scoring	N	Y
207	Sadideen, 2014 [103]	ND	ND	ND	N	N
208	Sadideen, 2016 [104]	ND	Two main themes were identified from post simulation. (1) participants felt the experience was authentic because the simulation had high psychological and social fidelity, and (2) there was a demand for TBS to be made readily available to improve nontechnical skills and interprofessional relations in burns and other emergencies.		N	N
209	Sagalowsky, 2018 [105]	Attitudes/Confidence /Knowledge	Attitudes, confidence and knowledge	5 likert scales	N	Y
210	Scalon da Costa, 2019 [106]	Knowledge/OSCE checklist	Cognitive knowledge test and the Objective Structured Clinical Examination (OSCE) checklist		Y	Y
212	Schmutz, 2014 [107]	ND	ND	ND	N	N
213	Schmutz, 2015 [108]	ND	ND	ND	N	N
214	Sepúveda Oviedo, 2022 [109]	ND	ND	ND	N	N
216	Seto, 2017 [110]	OSCE checklist	A multiple-choice question (MCQ) test, bag-mask ventilation (BMV) checklist, and two objective structured clinical examinations (OSCEs)	OSCE checklist	N	Y
218	Shin, 2014 [111]	ND	ND	ND	N	Y
220	Siebert, 2022 [112]	Number of errors, delay	<Primary outcome -total number of errors in first study>(a) correct pediatric pad size and anterior-posterior placement in the center of the exposed child’s chest +/-1 cm; (b) correct defibrillator operating mode; (c) adequate choice of energy dose (AHA recommendations for the arrhythmia being treated; (d) load of energy dose; (e) verbalization of the safety precaution measures before shock delivery; and (f) delivery of electric current < secondary outcome> (a)the total number of errors (b) delay (in second)	PALS checklist	Y	Y
221	Sigalet, 2012 [113]	Attitudes	ATTITUDES questionnaire (1) relevance of IPE, (2) relevance of simulation, (3) communication, (4) situation awareness, and (5) roles and responsibilities	Published already	N	Y
224	Smith, 2019 [114]	ND	ND	ND	N	N
228	Teis, R, 2017 [115]	Performance/Team performance	Primary outcomes (feasibility): number of success, Secondary outcomes:Resuscitation performance,Team performance: cardiac compressions, rate, depth, fully released, ventilation rate(%),	ND	N	N
229	Tobler, K, 2014 [116]	Confidence/ Performance	Self-assessment(confidence)/ performance(expert 2 + parent평가)	ND	N	Y
230	Tofil, N. M, 2017 [117]	Mental,Physical,Temporal demand/Performance/Effort/ Frustration	Mental demand, physical demand, temporal demand, performance, effort, frustration	ND	N	N
232	Traynor, 2021 [118]	Skills/ Critical thinking/ assessment	ICU nursing skills and critical thinking.Environmental and Safety Assessment,Physical Assessment,Critical Thinking	Published already	Y	Y
233	Tsai, T. C, 2003 [119]	Skills/Behavioral performance	Reliability (internal consistency, Cronbach’s a)	The scales for the task-specific skill checklist and the behaviour rating were dichotomous.	N	Y
235	Tyler, 2021 [120]	Performance	Performance dyadic subscale (PDS),Reflection dyadic subscale	ND	N	Y
238	Ventre, K. M., 2009 [121]	Knowledge/Performance	Each case was designed to test the particpant’s knowledge of the complete PALS treatment algorithm for that condition/Pilot Study of PALS Providers’Performance	AHA checklist	Y	Y
240	Wallace, 2010 [16]	Performance	ITC Sense of Presence Inventory,Social Attractiveness Questionnaire:	Published already	Y	Y
242	Walton, J. L, 2018 [122]	Assessment	Test Scores Before and After Educational Intervention/Average Scores for the Major Domains Assessed for Each of the Scenarios	ND	N	N
243	Watkins, S. C., 2021 [123]	Technical skills/Behavior performance/Team assessment	The TS assessment tools consisted of a scenario-specific checklist and a global rating scale (GRS)/Nontechnical Skills Rating Instruments-The TEAM tool(Team emergency assessment measure,BARs tool(Behavioral Anchored rating scale)	Technical Skill (TS), Behavior Anchored Rating Scale (BARS), TEAMS	Y	Y
245	Watkins, S. C, 2017 [124]	Skills/Behavioral performance	Anesthetists’ Nontechnical Skills (ANTS), BARS behaviorally anchored rating scale (BARS)		Y	Y
248	Whalen, A. M, 2018 [125]	ND	ND	ND	N	N
249	Whalen, A. M, 2022 [126]	Assessment	Assessment Tool Development	Published already	Y	Y
252	Naoko NAMBA, 2021 [127]	ND	ND	ND	N	N

ATV, All-Terrain-Vehicle; AHA, american heart association; BARS, behaviorally anchored rating scale; GIOSAT, generic integrated objective structured assessment tool; GRITS, global rating index for technical skills; KCS, knowledge and clinical skills; LCS, leadership and communication skills; ND, not described; OSATS, objective structured assessment of technical skills; OSCE, objective structured clinical examination; PACT, performance assessment tools for Interprofessional communication and teamwork; PALS, pediatric advanced life support; PBPQ, Pain Beliefs and Practices Questionnaire; TEAMS, team emergency assessment measure; TS, technical skills; VAS, visual analogue scale; VE, virtual environment.

## Discussion

SBE is recognized as an important field in health education [19], and its technology and field are being advanced and expanded at an astonishing pace [19]. In particular, the need for P-SBE is growing because pediatric patients require highly proficient skills, despite limited access by students in clinical settings [20]. In the present study, we conducted a systematic review to identify the characteristics of the P-SBE programs. We also examined the methods of validity and reliability testing in studies that developed the P-SBE programs. We aim to describe these topics based on the general characteristics of the research for discussion.

Navigating through the vast literature, a total of 142 studies on P-SBE were identified. While research in this field was limited prior to 2004 (n = 3, 2.1%), substantial research has been conducted from 2004 to the present (n = 139, 97.9%). In particular, there has been an increase in up to 20 studies since 2011. Simulations were introduced in medical and nursing education in the 1960s when mannequins that enable training of mouth-to-mouth breathing were developed; owing to advances in state-of-the-art technology and artificial intelligence, types of simulations, fields of application, and simulation scenarios have become increasingly similar to real-world situations, allowing for the achievement of special educational objectives [21]. Moreover, according to the IOM recommendation that education for healthcare providers must comprise evidence-based content and that new technology, such as team-based simulations, should be incorporated into the curriculum to provide safer and more effective treatment [22], SBE strategies are anticipated to be further expanded and advanced in the coming years.

Next, by country, there was the most active research in developed countries, including the United States, with 62 (43.7%) studies published in the United States, 17 (12.0%) studies in Canada, and 10 (7.0%) studies in the United Kingdom. This may be attributable to the fact that while national leaders, organizations, and accreditation bodies have spared no support from educators of healthcare providers in transforming the present and have served a central role in simulation education, SBE has advanced primarily around organizations such as the Society for Simulation in Healthcare (SSH) and International Nursing Association for Clinical Simulation and Learning (INACSL), which mostly includes developed countries [23]. In the future, education systems that provide P-SBE to healthcare providers should be expanded to countries with poor supportive networks.

Based on the study design, the most common type of study design was experimental, including RCTs (n = 33, 23.2%) and quasi-experimental studies (n = 18, 12.7%). The prominence of experimental designs emphasizes the scientific accuracy and commitment of the research community in producing evidence-based results in the field of P-SBE. The focus of current research mainly on the development and evaluation of simulation programs is a positive sign. This trend indicates the academic community values ensuring that P-SBE programs are not only innovative but also effective in delivering essential skills to healthcare providers. Even though such designs have been widely adopted, there is a need to consider mixed methods approaches in the future, capable of offering both quantitative data and deeper qualitative insights into learners’ experiences and perceptions. Additional research is necessary to assess not just the effectiveness but also the feasibility, accessibility, and scalability of P-SBE across diverse environments.

By simulation type, 92 studies used an advanced patient simulator and 52.5% used only a high-fidelity simulator. Next, 30 (32.6%) studies used computer-based training and 17 (20.2%) them used video-based simulations. Ten studies used a standardized patient (SP)/participant, and nine (6.3%) of them used an SP. This is because the key to simulation education for healthcare providers, which is defined as skills training, learning, assessment, testing or system, or platform for gaining an understanding of human behavior in a situation or environment that allows them to experience real-world cases [24], is how well it reflects reality, and high-fidelity simulators provide modifiable, realistic responses to the situation and learners’ input. The current level of technology allows high-fidelity simulators to precisely mimic human body functions and provide realistic responses, such as heart and lung sounds, chest movements, and detectable pulses, enabling learners to be integrated into patient scenarios that require their clinical judgment and practice proficiency [25]. Research utilizing VR or other games is rare. Such technology reflects real-world situations and can detect learners’ real-time responses to changes in the situation, but it is rarely used. In particular, the fact that 17 out of 30 (56.7%) studies on computer-based training used video-based simulations shows that this area requires further development.

The target audience for the scenario’s content could be determined through the analysis of the scenario itself. A total of 98 studies were included in the analysis of the content of P-SBE scenarios in Korea and other countries. Of the 44 studies that developed programs for medical staff, 37 (37.8%) were conducted with students, residents, and fellows. Thirteen (13.3%) studies were conducted on medical staff, nursing staff, and other staff, and 12 (12.2%) studies on other staff, including paramedics, lifeguards, and respiratory therapists. Several studies have developed programs for interdisciplinary teams. The core principle of healthcare providers is “First do not harm” [26]. Nevertheless, it has been reported that at least 44,000 (probably 98,000) patients die each year due to preventable errors by healthcare providers [27]. Simulation training enables the development and maintenance of skills in patient safety and quality management of medical services, and can help to acquire non-technical skills development and knowledge, such as communication skills and critical thinking, and to understand conceptual relationships [28] In addition, developing competencies related to interprofessional practice, including effective communication skills and teamwork, was recognized as essential to maximize patient outcomes and improve patient safety [29], confirming that the program was being developed for the team.

In terms of the five-stage model of skill acquisition [30], the most common stage targeted by SBE programs was competency (n = 29, 29.6%) and proficient (n = 29, 29.6%), followed by advanced beginner (n = 21, 21.4%), expert (n = 10, 10.2%), and novice (n = 9, 9.2%). In the 17th century, Dreyfus brothers developed a five-stage model to describe how individuals acquire skills and how experts master them. In other words, more studies have developed simulations designed to promote mastery among individuals at the competent or proficient level, which requires highly advanced and complex skills and experiences in more complex and challenging situations, as opposed to simulations targeting novices learning simple skills.

Subsequently, the scenarios were categorized based on their content. The most common scenario topic was emergency intervention (n = 59, 60.3%), and of these studies, there were 37 studies on pediatric rescue, 11 on neonatal rescue, and 8 on airway management. Another scenario was communication ability and decision making (n = 19, 22.4%), and the most common topic in this category was critical decisions (n = 13). This is in line with the Institute of Medicine (IOM) recommendations that healthcare providers are required to make accurate and critical decisions within a few seconds, even amid incomplete and inaccurate information; for these reasons, they must keep abreast with technological advances and collaborate with other professionals to rescue patients with complex morbidities [27]. Among programs targeting children, two studies developed a simulation program to enhance the decision-making ability of children with ASD, and programs targeting students included programs on resuscitation and decision-making ability during smoking education. These results show that SBE programs for children aim to improve their decision-making abilities. This is because simulation, an adaptive educational technology, provides an immersive environment in which students can interact with a given patient scenario and make their own decisions, through which they gain insight into their decision-making ability [31].

Regarding the reliability and validity of the scenarios, 30 studies (30.6%) tested the validity and 23 (23.5%) tested the reliability of the scenarios. In other words, there were still many studies that did not validate their findings despite the requirement for studies to be published to include evidence for evaluation or intervention, method of realization, reliability and validity, and educational outcomes to enhance the quality of evidence in medical education [32]. Reliability refers to the degree to which consistent measurements are obtained from the same study population. Validity refers to the degree to which something measures what it intends to measure. These crucial concepts underscore the need for more research to undertake such validation processes and reinforce their results, ensuring their applicability as trustworthy studies in a more effective manner.

Finally, in terms of the outcome variables used in the included studies, skills were the most common (n = 28, 28.6%), followed by performance (n = 24, 24.5%), knowledge (n = 17, 17.3%), and confidence (attitude) (n = 12, 12.2%). These results are in line with the recommendations of the (WHO) recommendations to develop standards and guidelines for simulation-based activities and implement simulation-based activities to accelerate the learning process and provide an opportunity for students and professionals to develop their skills and competencies [33].

In our systematic review, we examined the characteristics and development trends of P-SBE. Research in this domain was limited before 2004 but has witnessed significant growth post-2010. We observed that many P-SBE programs utilize high-fidelity simulators and team-based simulations, with emergency interventions being the primary educational topic to nurture rescue competencies. Most of this research has been conducted in developed countries like the United States, Canada, and the United Kingdom. While our results confirm the considerable advancement in P-SBE, many studies have not critically evaluated their validity and reliability. There’s a pressing need for an international protocol for the development of P-SBE, alongside rigorous validation and reliability testing. Furthermore, incorporating virtual reality technology could enhance the learning experience. It’s noteworthy to mention the limitations of our review: potential publication bias due to the focus on published papers, and the exclusion of scenarios where content specifics were not provided.

## Conclusion

SBE has become indispensable owing to strengthened patient rights and the growing importance of patient safety. SBE is an educational method that enables pediatric healthcare staff to effectively improve their proficiency and competencies. It provides an immersive environment in which learners can interact with the given patient case scenario and make decisions, and owing to such benefits, it is actively utilized to train attitude, knowledge, and skills in health care providers and other staff. We hope that studies continue to follow up on these programs and evaluate their validity and reliability. Furthermore, there is a need for instruments that enable the categorization of scenarios and simulations based on the objective and learner’s current level and assess their competencies by level.

## Data Availability

The datasets used and/or analyzed during the current study are available from the corresponding author on reasonable request.
